# Effect of Tailoring in an Internet-Based Intervention for Smoking Cessation: Randomized Controlled Trial

**DOI:** 10.2196/jmir.1605

**Published:** 2011-12-15

**Authors:** Silje C Wangberg, Olav Nilsen, Konstantinos Antypas, Inger Torhild Gram

**Affiliations:** ^1^Regional Centre on Substance AbuseUniversity Hospital of North NorwayNarvikNorway; ^2^Department of Clinical MedicineUniversity of TromsøTromsøNorway; ^3^Norwegian Centre of Integrated Care and TelemedicineUniversity Hospital of North NorwayTromsøNorway; ^4^Department of Community MedicineUniversity of TromsøTromsøNorway

**Keywords:** Smoking cessation, tailored feedback, email

## Abstract

**Background:**

Studies suggest that tailored materials are superior to nontailored materials in supporting health behavioral change. Several trials on tailored Internet-based interventions for smoking cessation have shown good effects. There have, however, been few attempts to isolate the effect of the tailoring component of an Internet-based intervention for smoking cessation and to compare it with the effectiveness of the other components.

**Objective:**

The study aim was to isolate the effect of tailored emails in an Internet-based intervention for smoking cessation by comparing two versions of the intervention, with and without tailored content.

**Methods:**

We conducted a two-arm, randomized controlled trial of the open and free Norwegian 12-month follow-up, fully automated Internet-based intervention for smoking cessation, slutta.no. We collected information online on demographics, smoking, self-efficacy, use of the website, and participant evaluation at enrollment and subsequently at 1, 3, and 12 months. Altogether, 2298 self-selected participants aged 16 years or older registered at the website between August 15, 2006 and December 7, 2007 and were randomly assigned to either a multicomponent, nontailored Internet-based intervention for smoking cessation (control) or a version of the same Internet-based intervention with tailored content delivered on the website and via email.

**Results:**

Of the randomly assigned participants, 116 (of 419, response rate = 27.7%) in the intervention group and 128 (of 428, response rate = 29.9%) in the control group had participated over the 12 months and responded at the end of follow-up. The 7-day intention-to-treat abstinence rate at 1 month was 15.2% (149/982) among those receiving the tailored intervention, compared with 9.4% (94/999) among those who received the nontailored intervention *(P* < .001). The corresponding figures at 3 months were 13.5% (122/902) and 9.4% (84/896, *P* =.006) and at 12 months were 11.2% (47/419) and 11.7% (50/428, *P* = .91). Likewise, the intervention group had higher self-efficacy and perceived tailoring at 1 and 3 months. Self-efficacy was found to partially mediate the effect of the intervention.

**Conclusion:**

Tailoring an Internet-based intervention for smoking cessation seems to increase the success rates in the short term, but not in the long term.

## Introduction

The Internet seems to provide a promising setting for combining the ability to reach a lot of smokers with good effectiveness with a low cost per smoker. In a recent Cochrane review of 20 randomized and quasi-randomized trials on Internet-based interventions for smoking cessation, Civljak and colleagues [1] concluded that some Internet-based interventions can assist smoking cessation. Interventions appropriately tailored to the users and with frequent automated contact seemed most promising, although the results were inconsistent. Shahab and McEwen [2] concluded in their meta-analysis of Internet-based interventions for smoking cessation that the tailored interventions increased 6-month abstinence rates by 17% (95% confidence interval [CI], 12%–21%) compared with the nontailored intervention.

A tailored intervention is one that is adapted to the characteristics of the individual, and it is typically based on responses to a questionnaire. The main ways of tailoring can be classified into personalization, adaptation, and feedback [3]. Personalization refers to making references to the recipient in the text such as first name, age, gender, or hometown. Adaptation concerns the content of the text itself, which can be tailored according to a variety of theories. Health psychological models often form the core of adaptive tailored interventions. Self-efficacy is one of the theoretical constructs that have shown the most consistent effects as a result of tailoring [4]. The third method of tailoring, feedback, is a widely used feature of tailoring in which the recipient is informed about scores on a scale and how the score can be interpreted. In recent, more complex tailoring, these features are often combined, and the components of the Internet-based intervention may also be tailored.

Although the literature suggests that tailoring is an important part of Internet-based interventions for supporting health behavioral change, we do not know how important it is compared with other components, such as discussion forums, personal quitting plans, and diaries, or how these components might interact. One way of studying these relationships is to compare a full intervention with a version where one of the components, such as tailoring, has been removed. Strecher et al [5] compared a tailored Internet-based intervention for smoking cessation with a nontailored Internet-based intervention and found that after 12 weeks, continuous abstinence rates (using the number of users who had logged on at least once as the denominator) were 22.8% in the tailored group compared with 18.1% (odds ratio = 1.34) in the nontailored group. Etter [6] compared two versions of the smoking-cessation program Stop-Tabac.ch, where the control group received an online report tailored to a number of variables, whereas the intervention group received a similar report that was somewhat targeted to a reasonable stage of change according to their smoking status, but otherwise fixed in terms of the tailoring variables (eg, self-efficacy was set as low for all and attitude toward smoking was set as positive for all). The result was a report that might have actually been tailored for some by chance, but not for all the participants. At the immediate follow-up 48 hours later, it was found that 12% (intention-to-treat [ITT]) of the smokers in both groups had made a 24-hour quit attempt. Plausible explanations of this lack of increased effect in the tailored group include an unclear control condition with targeting to stage of change and the potential for both actual and pseudotailoring, in addition to the very short follow-up time period.

We aimed to isolate the effect of tailored feedback in a multicomponent Internet-based intervention for smoking cessation through randomly allocating participants to one of two versions of our Internet-based intervention: one with tailored feedback, or one that was otherwise similar but without the tailored feedback. The purpose of the study was to examine, in a 12-month randomized controlled trial, whether the 7-day abstinence rates would differ between those receiving the tailored intervention and those who did not. We also wanted to explore whether tailoring would result in improved self-efficacy and more use of the website.

## Methods

### Study Design and Participants

The study was a two-arm, 12-month, randomized controlled, Internet-based trial with continuous recruitment and data collection. The allocation ratio was 1:1. The intervention arm in the trial received tailored messages in addition to the basic functionality of the Internet-based intervention for smoking cessation, while the control arm did not. Enrollment started on August 15, 2006 and ended December 7, 2007. The study was approved by the Regional Ethics Committee for North Norway (REK-NORD) and the Norwegian Privacy Ombudsman for Research. The randomized controlled trial was initiated before trial registration became customary in Norway, and therefore does not have a trial identification number.

**Figure 1:**
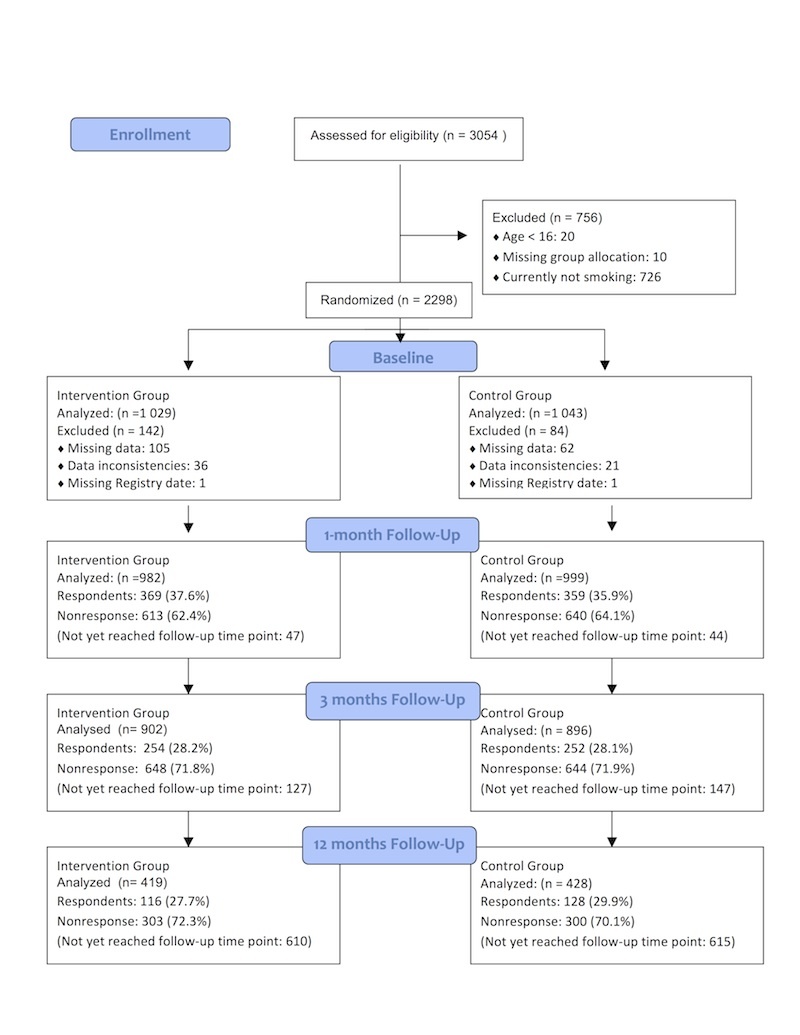
Participant flowchart.

The Intervention website was announced as a new and free service to aid in smoking cessation in the local and national media. All participants agreeing to the informed consent form were subsequently automatically allocated through use of an online random number generator to the intervention or control arm (for the informed consent form, see Multimedia Appendix 1). Altogether 3054 visitors registered to use the Norwegian website slutta.no. The front page displayed the logos of the Norwegian Directorate of Health’s Quitline, the Norwegian Cancer Society, and the Norwegian Centre for Telemedicine (for a screenshot of the front page of the intervention, see Multimedia Appendix 2). Registration required providing a unique email address, so potentially, using several email addresses, a person could have registered more than once. Among registrants, 30 were excluded (20 because they were under age 16 years and 10 because of missing group allocation). Another 726 registrants had already quit smoking and were excluded from the current analyses. Among the 2298 participants who smoked at enrollment, 1029 were randomly assigned to the intervention and 1043 to the control arm (Figure 1).

The participants filled in an extensive questionnaire at the time of enrollment. This included information on such demographic variables as gender, age, education, and work situation. The participants also provided a quit date and an email address. Further, they completed a smoking-cessation maintenance self-efficacy questionnaire, reported on smoking behavior, and stated their motivation for cessation. The tailored messages were created on the basis of these questionnaires and were sent to the intervention group on their personal webpage and by email (for a screenshot of My Page, see Multimedia Appendix 2). Participants in the control group did not get any messages on their webpage and only emails containing notifications and reminders for the follow-up questionnaires.

Both arms received an email message with a link to a questionnaire on self-efficacy and smoking behavior at 1, 3, and 12 months after the date of enrollment. On completion of the questionnaire, the participants could enter a draw to win prizes such as books and T-shirts. Nonresponders received up to three reminders.

### Internet-Based Intervention for Smoking Cessation (slutta.no): Basic Functionality

The intervention consisted of multiple intervention components and was intended for long-term follow-up. The website included static information on the dangers of smoking, general advice on smoking cessation, and information about the website. In addition there were interactive tests for nicotine addiction, type of smoker (stress smoker, comfort smoker, etc), and motivation level.

There was an emphasis on creating opportunities for social interaction using a discussion forum, a guestbook, and a personal diary (Multimedia Appendix 3). The participants could invite friends and other participants to support them by leaving messages in the guestbook, and to publish their own smoking-cessation diary. There were also some community features: participants could click on other participants’ nicknames in the forum and thereby get a specific profile with some information about the other participant, for example. The possibilities to interact were only as described above, as there were no opportunities for synchronous communication through chat or private messaging between the participants.

### Tailoring

During the 12-month intervention, the participants in the intervention group received up to 150 tailored messages. The first message was sent 14 days before, and the last, 12 months after, the quit date. The frequency of the messages varied over the course of the 12 months, as they were sent out on a defined number of days before or after the quit date. In the beginning messages were sent daily, then the frequency was decreasing slowly during the first 3 months with a substantial drop-off 3 months after the quit date.

The tailoring was set up on the basis of several different types of variables. Personalization-, adaption-, and feedback-type tailoring were all used to varying degrees. Table 1 lists examples of variables for tailoring. The self-efficacy messages were more specifically about confidence in refraining from relapsing in different situations, also known as maintenance self-efficacy [7]. In concordance with several stage and process models of health behavioral change, such as the Health Action Process Approach [7], we aimed at providing these as preparation to transition from conscious behavioral change (action) to lifestyle integration (maintenance). In this intervention we did not assess where participants were in their process through a questionnaire, but we did send maintenance self-efficacy messages to those with a low maintenance self-efficacy at 3 months past their quit date. There was no other tailoring based on a health psychological stage or process approach in the current intervention. Besides the messages concerning addiction, the rest concerning benefits of quitting smoking, social support, etc, were evenly distributed over the year, with decreasing frequency.

The tailored messages could also be retrieved from a calendar on the participant’s My Page. Other tailoring features on this page included a personalized greeting, feedback on number of smoke-free days and the amount of money saved, and a list of the reasons the participant had entered for wanting to quit smoking.

**Table 1:** Examples of the tailoring the participants in the intervention group received

Variable	Question	Answer example	Message example (sent relative to quit date)
Personalization	What would you like us to call you?	Jane	+365 days: Congratulations, Jane! Today you have been smoke-free for a year!
Quit date	When do you intend to quit?	[Date]	+5 days: There is no longer nicotine present in your body.
Step-down	Would you like to do a step-down of your smoking?	Yes, through smoke-free zones	–10 days: Create a smoke-free room in your home.
Self-efficacy	How confident do you feel about refraining from smoking when angry or upset?	1 = Not confident at all	Immediately on screen: Try to calm down instead of smoking when angry or upset. Relaxation techniques are one effective way to do it, and can be done quickly and discreetly, wherever and whenever, once you have practiced them. Another method is distraction. You can take a walk, read the paper, or play a game.
Main occupation	Are you currently working?	Yes, Working full time	–2 days: Consider which situations at work tempt you to smoke.
Social pressure	Do your friends smoke?	Yes, all of them	+58 days: Watch out! Some might like it if you fail. It could make them feel better.
Motivation	What is your most important reason for quitting?	I want to save money	+71 days: Try to calculate how much money you have saved. It might make you proud!
Social support	Would you like to tell others that you are quitting smoking?	Yes	–13 days: Tell your friends and family that you plan to quit.

### Measures

Data were gathered on age, gender, education, perceived tailoring, perceived usefulness of the website, self-efficacy, and smoking. Education was rated on a 5-point scale: 1, ≤9 years of total education; 2, 10–11 years; 3, 12 years; 4, 13–16 years; and 5, ≥17 years. Motivation was assessed with a single question, “How strong is your motivation for quitting smoking?” The participant answered on a 4-point scale ranging from “very weak” to “very strong.” Previous research has found that a similar single-item measure for motivation had comparable predictive validity to a multi-item instrument [8].

Data on the use of the interventions were gathered through Web logging. The number of log-ins and time spent at the site (in minutes) per user were registered. At the 1-month follow-up, the participants were asked whether they would recommend the site to a friend and to rate from a list of intervention components the one that they found the most useful.

Smoking behavior was assessed at the baseline and at 1-, 3-, and 12-month follow-ups as 7-day abstinence rates through the question “Have you during the last 7 days had a smoke, even just a single puff?”

Data on smoking-cessation maintenance self-efficacy were gathered at baseline and after use of the site for 1 month and 3 months, using the 12-item Smoking Self-Efficacy Questionnaire [9,10]. The 12 items consist of 6 items addressing perceived ability to refrain from smoking in the face of internal stimuli such as when nervous, and another 6 items addressing external stimuli such as when with other smokers. The scale has previously been shown to predict smoking cessation, to be negatively related to number of cigarettes smoked, and to have the ability to discriminate between stages of change [10]. The same study [10] found that the internal consistency was alpha = .94 for the internal subscale and alpha = .89 for the external. At baseline the items were rated on a 5-point scale that was later recoded into a 6-point scale (2 = 2.25, 3 = 3.5, 4 = 4.75, 5 = 6, ELSE = Copy), and on a 6-point scale at 1- and 3-month follow-up (1 = not confident at all, to 6 = completely confident). This recoding was necessary because in the tailoring questionnaires, which provided the baseline data, we used the original 5-point response format of the SEQ-12 [9] while, for purposes of conformity of response format in the evaluation questionnaires, we used a 6-point scale here.

Perceived tailoring was assessed with 4 items from Dijkstra [3] evaluating to what extent the user feels that the information is adapted to his or her personal situation. Agreement with these 4 items was rated on a 6-point scale ranging from 1, completely disagree, to 6, totally agree.

### Statistical Analyses

We based our a priori sample size estimation on a paper by Strecher [11], pointing out that previous computer-based smoking-cessation intervention studies have found group differences in abstinence rates of about 2%. Using abstinence rates at 12 months that only slightly exceeded no intervention (6% and 8%) and a 1-sided test without continuity correction at a .05 alpha level and with 90% power, we needed a total sample of 2787. Also, we expected to have to raise the number of participants recruited further by 40%–60%, that is, to around 4000, because of the high dropout often observed in Internet-based interventions [12].

No items had more than 5% missing data at the baseline; we therefore assumed missing data to be missing completely at random. On the variables *s* *elf-efficacy* and *p* *erceived tailoring*, we replaced the missing data with values imputed by the expectation maximum likelihood algorithm in SPSS version 16.0 (IBM Corporation, Somers, NY, USA) before analysis of variance. Internal consistency was measured by Cronbach alpha. Nonresponse on 7-day abstinence was dealt with by counting all participants with missing data as smokers (ITT). We compared the ITT quit rates with the quit rates for responders only.

Differences in dichotomous baseline characteristics and in abstinence rates between groups at all time points were analyzed with a regular chi-square test. Group differences in continuous variables were analyzed with *t* test. The Mann-Whitney *U* test was used for comparing the usage of the intervention between groups, as these distributions were nonnormal. Effect sizes for group differences at the different time points were calculated as relative risk.

Mediation was tested using an approach developed by Preacher and Hayes [13] using their SPSS macro [14]. Bootstrapping (5000 samples) with bias correction and acceleration was used to create a 95% CI around the point estimate of the indirect effect, with an interval not including zero indicating a significant indirect effect.

## Results

### Participant Characteristics and Response Rates

Both recruitment and data collection were continuous, and were maintained right until the point of data extraction. This implies that at the time of data extraction, some users of the intervention had been followed up for a few days and others for the full 12 months. In Figure 1 those participants not having had the possibility to reach the next follow-up time point are indicated as “Not yet reached follow-up time point,” while the true nonresponders are indicated by “Nonresponse”. The overall response rate was 36.8% (728/1981) after 1 month, 28.1% (506/1798) after 3 months, and 28.8% (244/847) after 12 months. There were no significant differences in response rates between the two groups at any time point (1 month: χ^2^_1_ = 0.58, *P* = .45; 3 months: χ^2^_1_ < 0.001, *P* = .99; 12 months: χ^2^_1_ = 0.51, *P* = .48).

Overall among the participants, 72.26% (1497/2072) were female, mean age was 37 years, 17.1% (353/2072) had 17 or more years of education, mean motivation score was 3 (range 1–4), mean self-efficacy score was 34 (range 0–60), mean number of cigarettes smoked per day was 16.

As can be seen in Table 2, there were no significant differences between the intervention and the control group at baseline. Furthermore, no group differences in demographics were found at the follow-up time points.

**Table 2:** Baseline comparisons

		Intervention group (n = 1029)	Control group (n = 1043)	Test statistic	*P* value
**Female**				χ^2^_1_ = 1.37	.24
	n (%)	732 (71.1%)	766 (73.4%)		
	95% CI^a^	68.3%–73.8%	70.8%–76.1%		
**Age (years)**				*t*_2013_ = 0.94	.35
	Mean	37.3	36.9		
	95% CI	36.7–38.0	36.2–37.5		
	Range	16–71	16–68		
**Education (years)**				χ^2^_4_ = 3.22	.52
	≤9, n (%)	51 (5%)	54 (5.2%)		
	95% CI	3.6%–6.4%	3.2%–6.5%		
	10–11, n (%)	157 (15.3%)	188 (18%)		
	95% CI	13.2%–17.5%	15.5%–20.5%		
	12, n (%)	188 (18.3%)	190 (18.2%)		
	95% CI	15.9%–20.7%	15.9%–20.5%		
	13–16, n (%)	455 (44.2%)	436 (41.8%)		
	95% CI	41.3%–47.2%	39%–44.7%		
	≥17, n (%)	178 (17.3%)	175 (16.8%)		
	95% CI	14.9%–19.8%	14.5%–19%		
**Occupational status^b^**				χ^2^_5_ = 1.78	.88
	Full-time employment, n (%)	610 (59.3%)	634 (60.8%)		
	95% CI	56.4%–62.2%	57.8%–63.7%		
	Part-time employment, n (%)	135 (13.1%)	130 (12.5%)		
	95% CI	11.2%–15.4%	10.5%–14.4%		
	Unemployed, n (%)	36 (3.5%)	28 (2.7%)		
	95% CI	2.4%–4.7%	1.7%–3.7%		
	Student, n (%)	149 (14.5%)	148 (14.2%)		
	95% CI	12.3%–16.6%	12.1%–16.1%		
	Retired, n (%)	27 (2.6%)	25 (2.4%)		
	95% CI	1.7%–3.7%	1.5%–3.5%		
**Cigarettes per day**				*t*_2064_ = –0.29	.77
	Mean	16.1	16.2		
	95% CI	15.6–16.5	15.7–16.6		
**Living with someone**				χ^2^_1_ = 4.22	.04
	n (%)	797 (77.5%)	846 (81.1%)		
	95% CI	74.9%–80.1%	78.8%–83.6%		

**Motivation score**				*t*_2069_ = 0.87	.38
	Mean	2.96	2.93		
	95% CI	2.91–3.00	2.89–2.97		
**Self-efficacy score**				*t*_2064_ = 1.36	.17
	Mean	32.6	32		
	95% CI	32–33.2	31.4–32.6		

^a^ Confidence interval.

^b^ There was also an ”Other” category not shown in the table.

### Use of the Intervention

Table 3 displays the time spent on different activities at the website according to study arm. The intervention group had logged on more times *(* *P =* .03) and had used the site more overall *(* *P* = .02). In more detail, the intervention group had used My Page more *(* *P* = .03) than the control group had. The most used component of the intervention was the discussion forum, followed by My Page, while the Facts section was used much less. More detailed analyses on the use of the intervention over time can be found in Wangberg et al [15].

**Table 3:** Number of log-ins and minutes of use overall for some of the core components of the intervention by group

	Group	Median	IQR^a^	*Z* score	*P* value
Number of log-ins overall	Intervention (n = 1029)	3	5		
	Control (n = 1043)	2	4	4.54	<.001
Minutes spent at site overall	Intervention	93	159		
	Control	68	107	5.46	<.001
Minutes spent in discussion forum	Intervention	6	27.5		
	Control	6	29	0.92	.36
Minutes spent at My Page	Intervention	7	13		
	Control	6	9	2.21	.027
Minutes spent reading Facts	Intervention	0	1		
	Control	0	1	3.33	.001

^a^ Interquartile range is a measure of variation for the median, which equals the difference between the third and the first quartile.

### User Evaluation

In the intervention group, 88.4% (320/362, 95% CI, 84.7–91.3) of the users stated that they would recommend the site to a friend, compared with 71.8% (255/355, 95% CI, 66.9–76.3, *P* < .001) in the control group. Further, in the intervention group, 34.0% (123/362, 95% CI, 29.3–39.0) of the users ranked the tailored emails as the most useful intervention component, compared with 6% (21/355, 95% CI, 3.9–8.9, *P* < .001) in the control group (who did not receive any emails besides one with username and password upon registration and emails with links to follow-up questionnaires). In the intervention group, 10% (37/362, 95% CI, 7.5–13.8) of the users ranked general information as the most useful component, compared with 22% (79/355, 95% CI, 18.2–26.9, *P* < .001) in the control group, while 15% (55/362, 95% CI, 11.9–19.3) of the users in the intervention group ranked the discussion forum as the most useful component, compared with 21% (73/355, 95% CI, 16.7–25.1, *P* = .06) in the control group. The remaining nominations were evenly spread over the 10 other functions the user could choose as the most useful.

### Manipulation Check: Perceived Tailoring

The *p* *erceived tailoring* scale was found to have good internal consistency at 1-month (alpha = .92) and 3-month (alpha = .94) follow-up. Table 4 shows mean scores on perceived tailoring by group at 1- and 3-month follow-ups. The intervention group had higher perceived tailoring scores at both time points (*P*s < .001).

**Table 4:** Perceived tailoring scores by group at follow-up

Time point		Intervention group	Control group	*t* test	*P* value
**1 month**				*t*_715_ = 4.50	<.001
	Mean	15.91	14.22		
	95% CI^a^	15.45–16.40	13.68–14.79		
	n	369	359		
**3 months**				*t*_502_ = 4.59	<.001
	Mean	15.45	13.37		
	95% CI^a^	14.85–16.09	12.72–14.04		
	n	254	252		

^a^ Confidence interval.

### Smoking Cessation

Table 5 shows that the ITT 7-day abstinence rate at 1 month was 15.2% (149/982) among those receiving the tailored intervention, compared with 9% (94/999) among those who did not (*P* < .001). The corresponding figures at 3 months were 13.5% (122/902) and 9% (84/896, *P* =.006) and at 12 months were 11% (47/419) and 12% (50/428, *P* = .91). The same group differences were found looking at responders only (Table 5).

**Table 5:** Group 7-day abstinence rates

Analytic strategy	Time point	Intervention group		Control group				
		Percentage (n/total)	95% CI^a^	Percentage (n/total)	95% CI^a^	χ^2^_1_	*P* value	RR^b^ (95% CI^a^)
All nonresponders counted as smokers (intention-to-treat)	1 month	15.2% (149/982)	13.1–17.6	9% (94/999)	7.8–11.4	15.3	<.001	1.61 (1.27–2.06)
	3 months	13.5% (122/902)	11.5–15.9	9% (84/896)	7.6–11.5	7.6	.006	1.44 (1.11–1.87)
	12 months	11% (47/419)	8.5–14.6	12% (50/428)	9.0–15.1	0.05	.91	0.96 (0.66–1.40)
Responders only	1 month	40.4% (149/369)	35.4–45.4	26% (94/359)	21.6–30.8	16.5	<.001	1.54 (1.25–1.91)
	3 months	48.0% (122/254)	41.9–54.2	33% (84/252)	27.5–39.2	11.3	.001	1.44 (1.16–1.79)
	12 months	41% (47/116)	31.5–49.6	39% (50/128)	30.5–47.6	0.1	.82	1.03 (0.76–1.41)

^a^ Confidence interval.

^b^ Relative risk.

### Secondary Outcome: Self-efficacy

Both the internal (alpha = .93) and the external (alpha = .86) self-efficacy subscales were found to have good internal consistency at 1-month follow-up. Table 6 shows the mean scores for self-efficacy at all follow-up time points. Self-efficacy was higher for the intervention group at 1- (*P* = .01) and 3-month (*P* = .002) follow-ups, but not after 1 year (*P* = .58), paralleling the results for the main outcome.

**Table 6:** Self-efficacy score by group at follow-up

Time point		Intervention group	Control group	*t* test	*P* value
**1 month**				*t*_626_ = 3.60	.01
	Mean	41.57	38.36		
	95% CI^a^	40.42–42.71	37.03–39.70		
	n	369	359		
**3 months**				*t*_336_ = 3.15	.002
	Mean	42.45	38.69		
	95% CI^a^	40.82–43.98	36.98–40.38		
	n	254	252		
**12 months**				*t*_211_ = 0.51	.58
	Mean	39.59	38.60		
	95% CI^a^	36.96–42.24	35.79–41.57		
	n	116	128		

^a^ Confidence interval.

### Test of Mediation: Self-efficacy and Perceived Tailoring

We performed a mediational analysis (n = 386) with group as the independent variable, 7-day abstinence at 3 months as the dependent variable, and self-efficacy and perceived tailoring at 1 month as the proposed mediators. The total effect of group on abstinence rates at 3-month follow-up was 0.66 (Wald χ^2^_1_ = 9.82, *P* = .002). Self-efficacy accounted for an indirect effect of 0.33 (95% CI, 0.08–0.60), while perceived tailoring did not have a significant indirect effect (point estimate = 0.003, 95% CI, –0.09 to 0.10). The remaining direct effect of group on abstinence rates was 0.58 (Wald χ^2^_1_ = 5.82, *P* = .02).

## Discussion

The results show that both 7-day abstinence rates and self-efficacy for smoking cessation were higher among those in the tailored intervention group at 1- and 3-month follow-ups, but not at the 12-month follow-up. The short-term results are consistent with previous studies [2].

We found that the intervention group had used the intervention more. One of the ways that tailoring may lead to higher smoking-cessation success is through providing a higher dose of the intervention. A previous study has shown that tailored emails increased adherence to the same Internet-based smoking-cessation intervention, but only up until 5 months [15]. Simple dose–response relationships have been found previously [16-18], but Danaher and colleagues [19] did not find a mediational effect of program exposure when controlling for self-efficacy, suggesting that the issue is not as simple as mere quantity of exposure to the intervention. Like Danaher and colleagues [19], we also found that self-efficacy partially mediated the effect of the intervention.

More participants in the intervention group than in the control group would recommend the intervention to a friend, with the tailored emails being ranked as the most useful feature of the intervention. In comparison, the participants in the control group (who did not receive the tailored emails) found the generic information and the discussion forum to be the most useful features.

The intervention group, which was the only one receiving tailored content, reported higher scores on perceived tailoring at 1- and 3-month follow-ups. We did not find that perceived tailoring mediated the effect of the intervention. Perceived program relevance (and amount of the materials read) have previously been found to mediate the effect of a tailored Internet-based smoking-cessation program [20], and an experimental study has even shown that perceived tailoring can account for the effect in a placebo tailored condition [21]. This was further supported by a later study where Webb and colleagues [22] were able to increase the effect of tailoring further by using expectancy (that tailored content is superior to generic) priming.

The main strengths of the current Internet-based smoking-cessation trial were a high sample size and repeated measurement. As this was an effectiveness trial, the results have higher external validity. Only age, access to the Internet, and willingness to set a quit date during the next 3 months were inclusion criteria for the present study, thus providing more relevant information for implementation in a real-world setting outside of strongly controlled clinical trials. At the same time, however, the representativeness of the study was compromised by the fact that the sample was self-selected. The results, thus, cannot be generalized to all people pursuing smoking cessation. Especially, the findings are less generalizable to men, since women in this study, as in previous ones [2], were overrepresented. Women generally tend to use the Internet more for health purposes than men do, and possible reasons for this include women’s traditional caretaking role and greater preferences for social support [23]. Our sample also had a relatively high educational attainment, and we are currently running a trial (clinical trial #NCT011030427) on whether the use of short message service (SMS) can increase use of the intervention by those with lower educational attainment. A study we did on delivering diabetes information via SMS suggested that the short format and push delivery might increase attention to and comprehension of the information [24].

A limitation of our study was that we were not able to separate receiving tailored content from receiving emails per se. Another limitation that this study shares with many other Internet-based interventions [14,25] is a high attrition and, thus, low response rate at follow-up assessments. It is likely that some of the participants through interactions in the discussion forum noted that they had not received the “full” version—for example, did not receive any advice by email. Despite this, we did not find differential attrition, and reached similar conclusions concerning the main outcome whether we used the ITT strategy of counting all nonresponders as smoking or analyzed just the responders. A follow-up study of nonresponders to a quitline study indicates that the ITT yields too low actual quit rates, as many of the people they followed up were (still) abstinent [26].

Furthermore, as seen from the records of website use in this study, and in Internet-based interventions generally, the consistency of delivery is often high, although the amount of time spent with the intervention can vary greatly between participants, with some of them barely visiting the site at all, as also seen in previous research [25,27-29].

### Conclusions

This randomized controlled trial found that tailoring an Internet-based intervention for smoking cessation increases success rates in the short term, but not in the long term.

## Multimedia Appendix 1

Informed consent form for the intervention Opptur/www.slutta.no. (Translated from Norwegian).

## Multimedia Appendix 2

Screenshot of the front page of the intervention Opptur/www.slutta.no.

## Multimedia Appendix 3

Screenshot of "My Page" where, among other functionality such as a diary and a guest book, tailored feedback was displayed on the intervention Opptur/www.slutta.no.

## Abbreviations

CI: confidence interval
ITT: intention-to-treat
SMS: short message service
