# Supplementary figures and images for: Effect of Tailoring in an Internet-Based Intervention for Smoking Cessation: Randomized Controlled Trial
Source: J Med Internet Res. 2011 Dec 15;13(4):e121. doi: 10.2196/jmir.1605 (PMC3278107; doi:10.2196/jmir.1605)

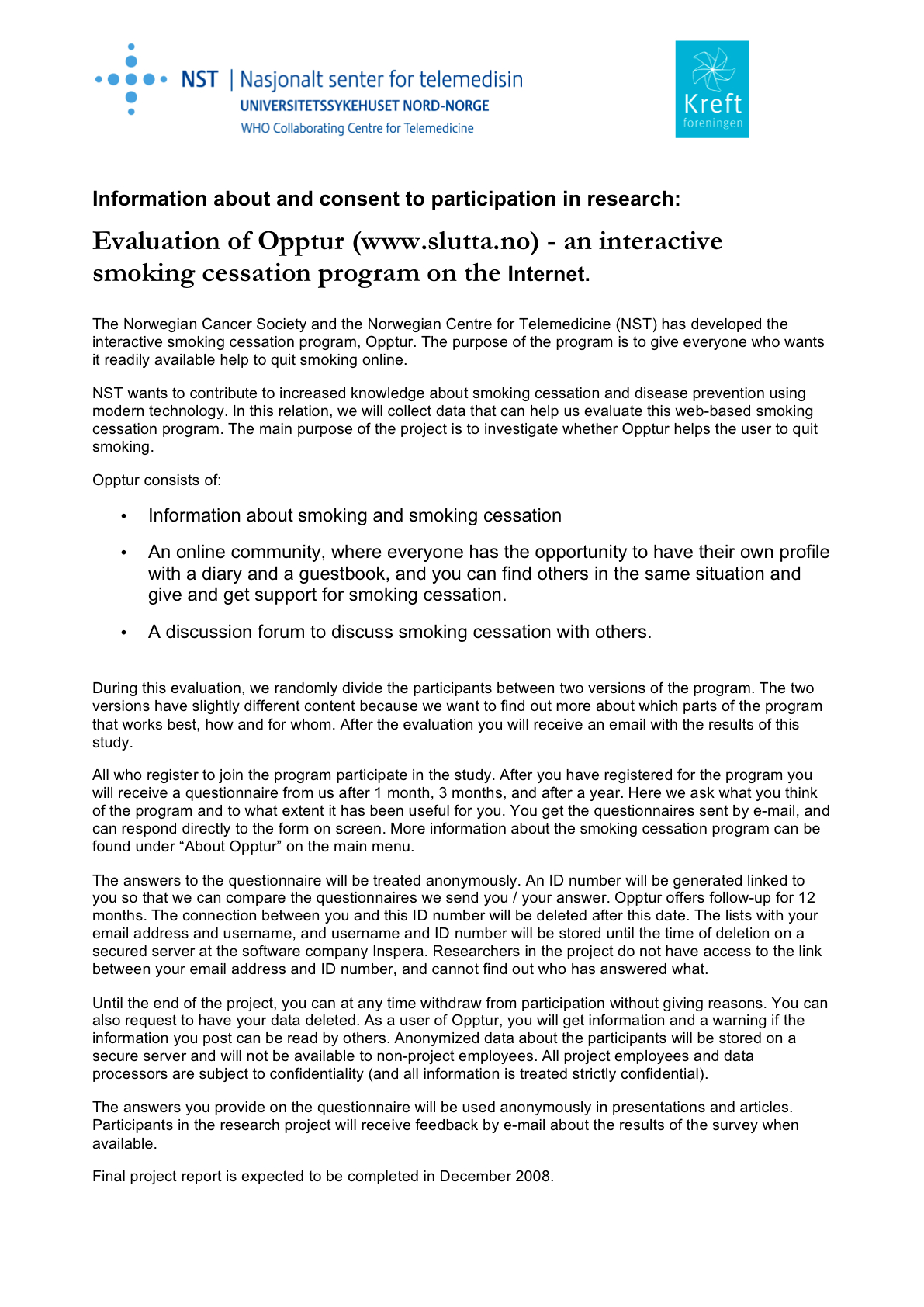

Supplement: Supplementary file 1 [file jmir_v13i4e121_app1.jpg]

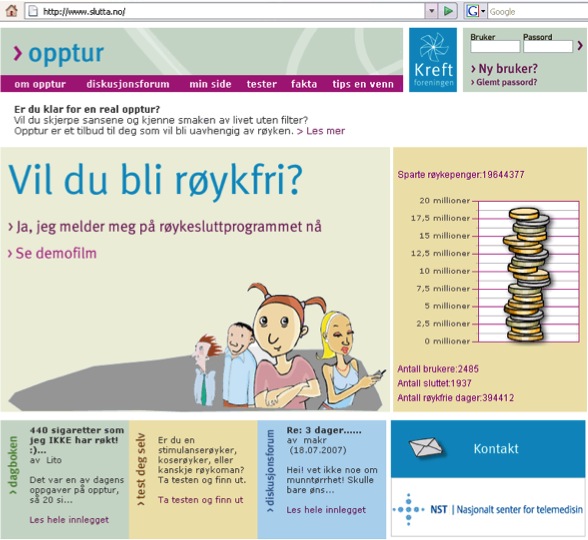

Supplement: Supplementary file 2 [file jmir_v13i4e121_app2.jpg]

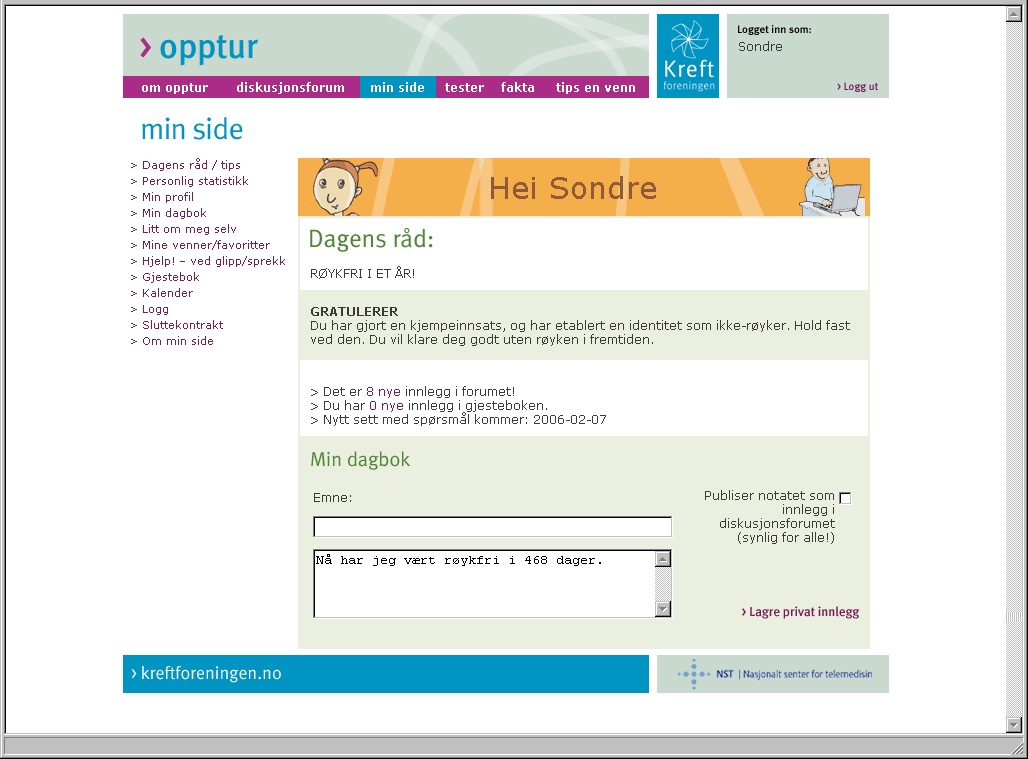

Supplement: Supplementary file 3 [file jmir_v13i4e121_app3.jpg]
